# Supplementary figures and images for: Dual function of the PI3K-Akt-mTORC1 axis in myelination of the peripheral nervous system
Source: eLife. 2017 Sep 7;6:e29241. doi: 10.7554/eLife.29241 (PMC5589416; doi:10.7554/eLife.29241)

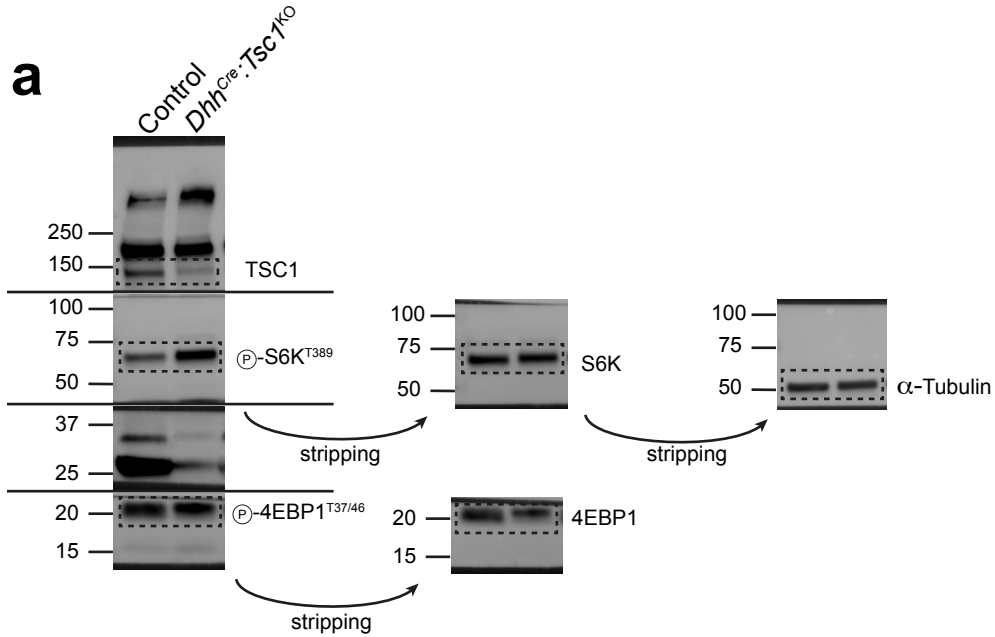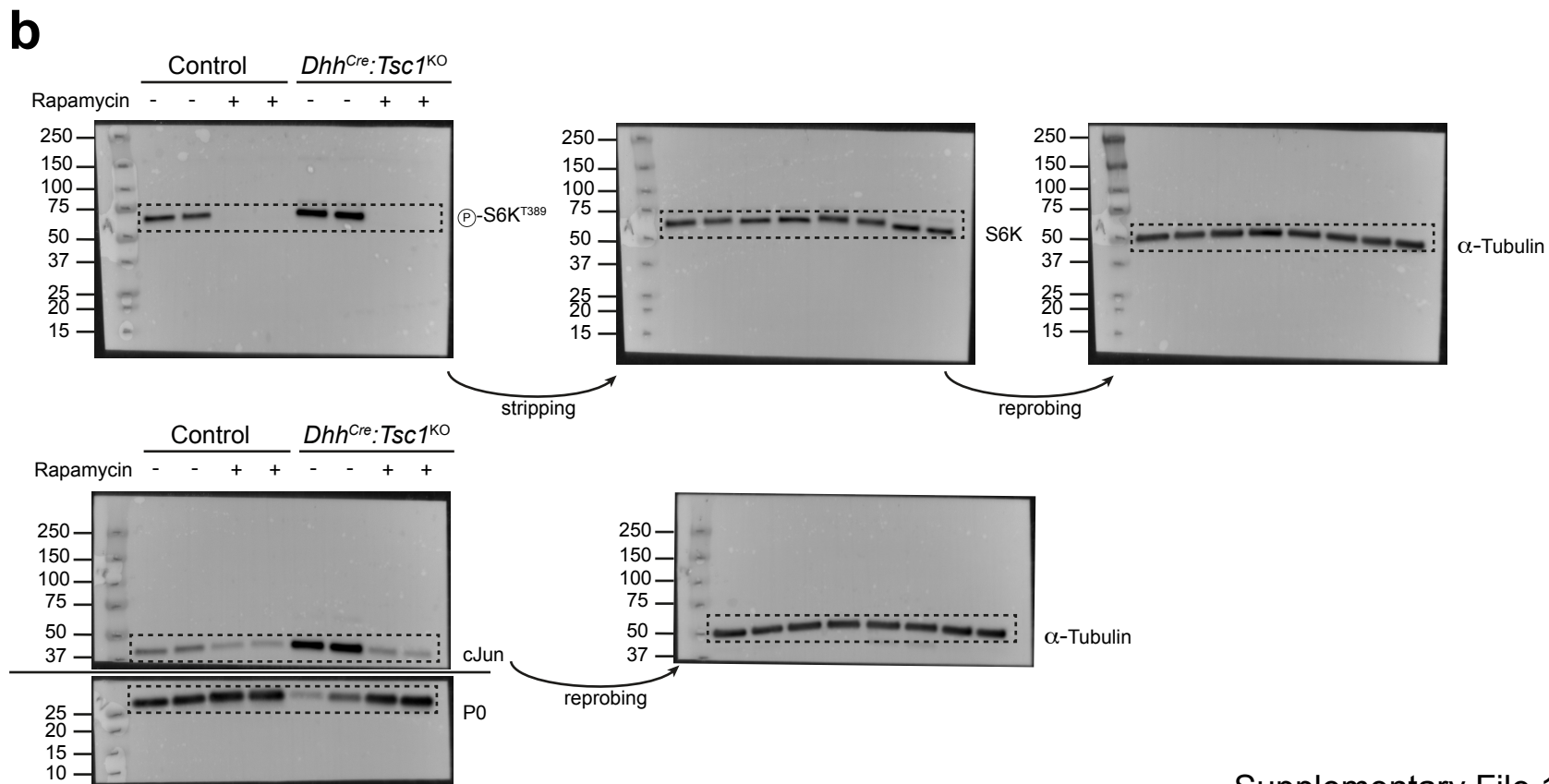

Supplement: Supplementary file 1. — (a) Western blot in Figure 1a. The membrane was cut as indicated by the continuous line and probed with the indicated antibodies. (b) Western blot in Figure 1k. The membrane was cut as indicated by the continuous line and probed with the indicated antibodies. [file elife-29241-supp1.pdf]

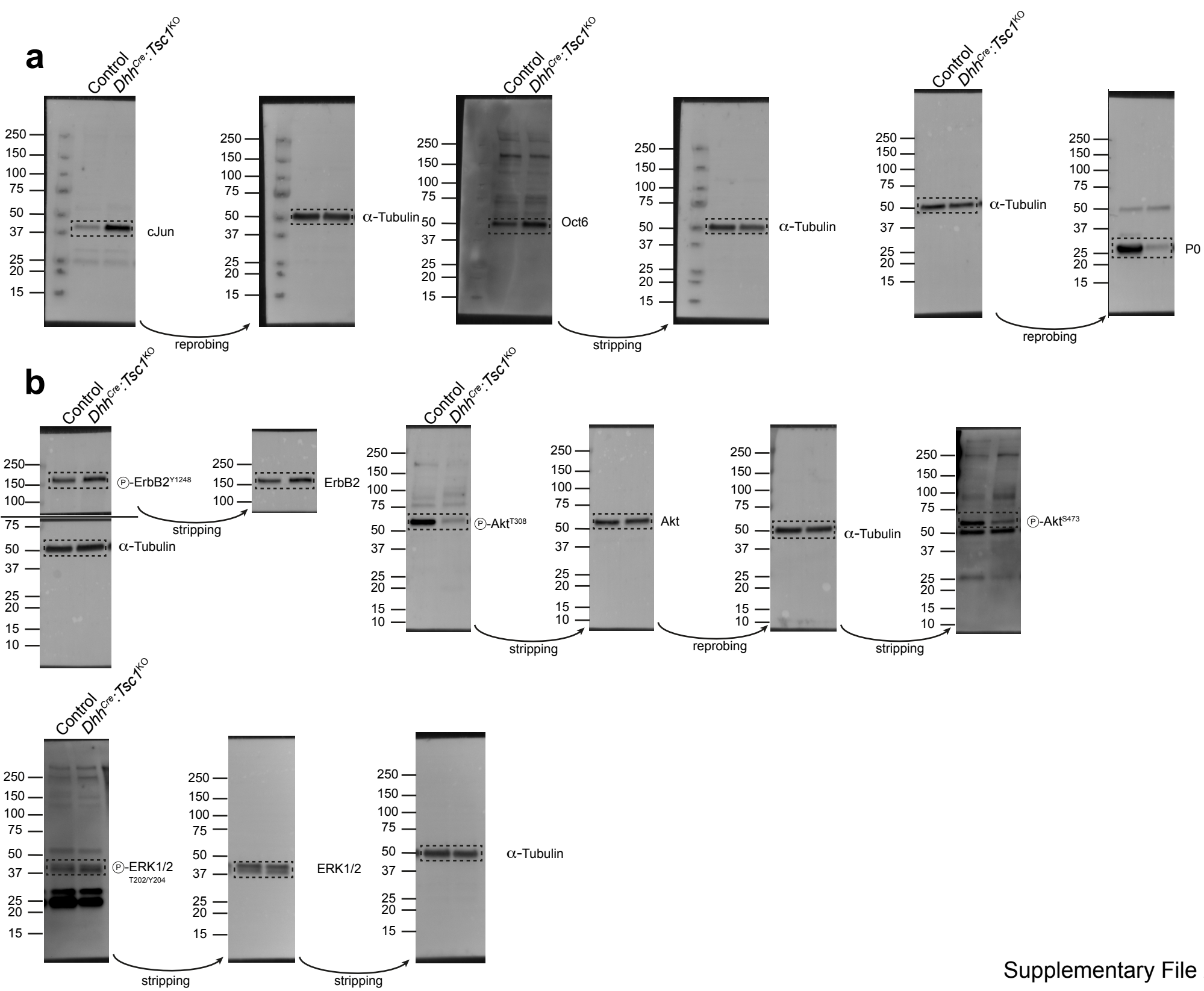

Supplement: Supplementary file 2. — (a) Western blot in Figure 1—figure supplement 2c. (b) Western blot in Figure 2a. The membrane was cut as indicated by the continuous line and probed with the indicated antibodies. [file elife-29241-supp2.pdf]

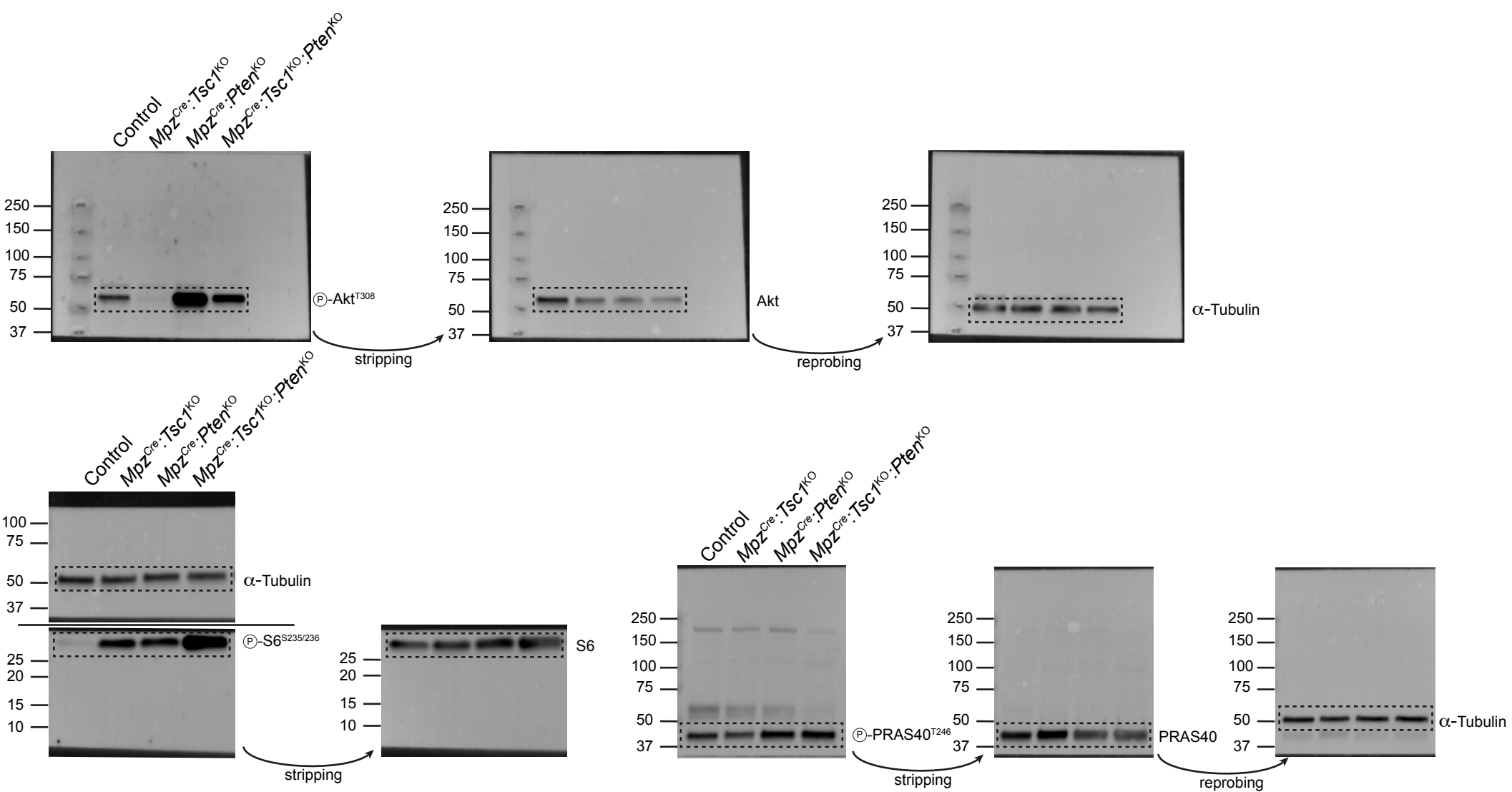

Supplement: Supplementary file 3. — Western blot in Figure 2c. The membrane was cut as indicated by the continuous line and probed with the indicated antibodies. [file elife-29241-supp3.pdf]

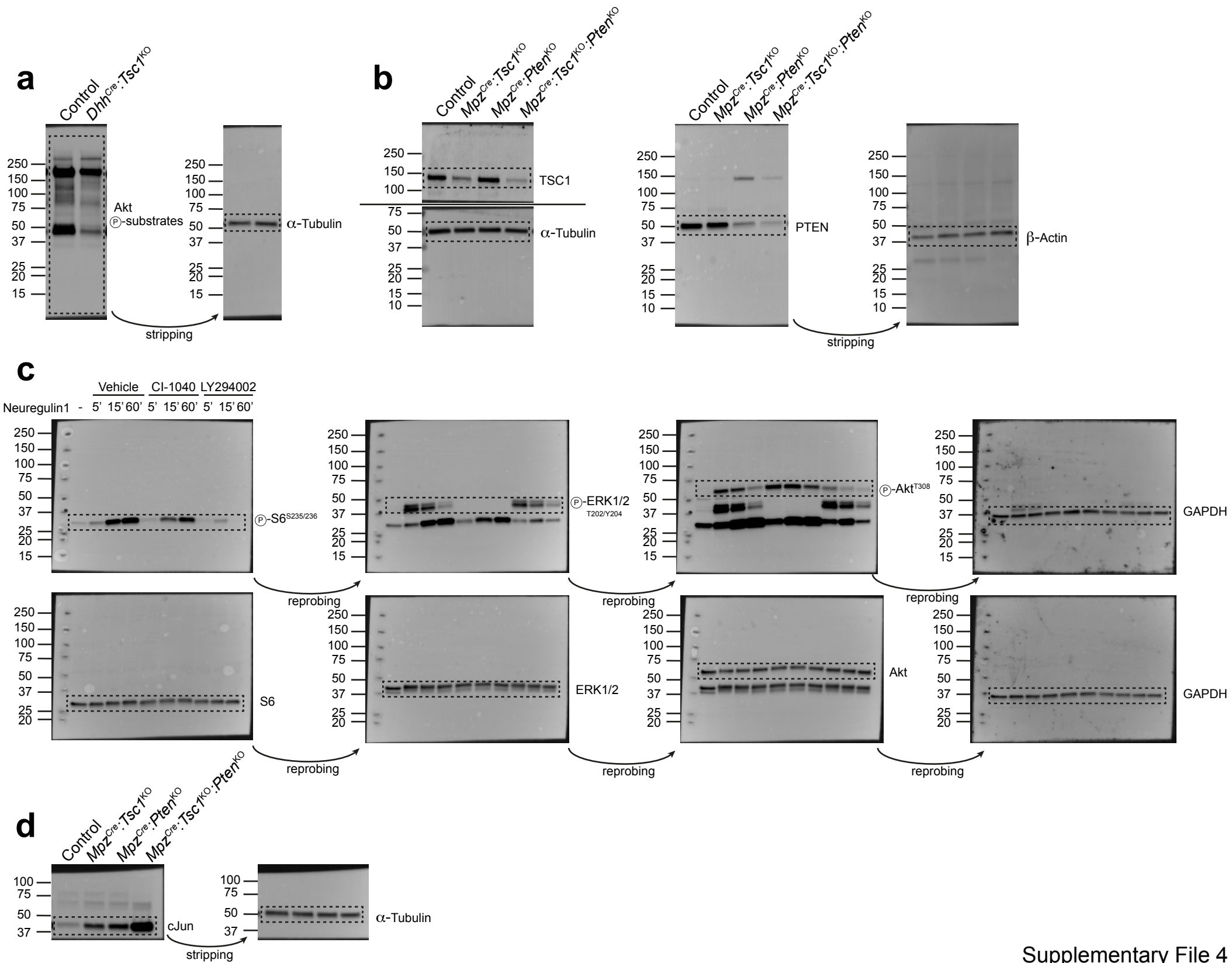

Supplement: Supplementary file 4. — (a) Western blot in Figure 2—figure supplement 2a. (b) Western blot in Figure 2—figure supplement 2c. The membrane was cut as indicated by the continuous line and probed with the indicated antibodies. (c) Western blot in Figure 2—figure supplement 2b. (d) Western blot in Figure 2g. [file elife-29241-supp4.pdf]

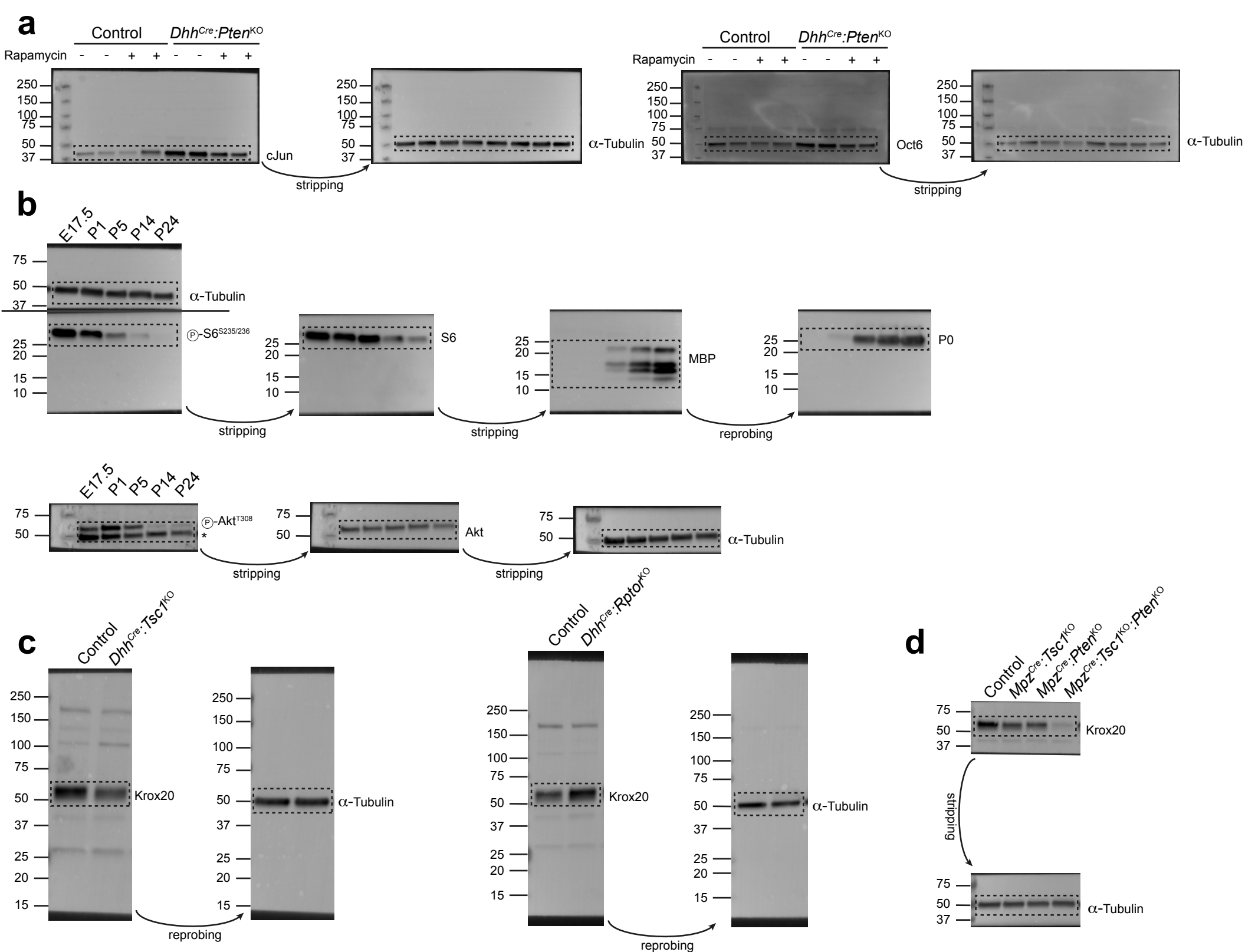

Supplement: Supplementary file 5. — (a) Western blot in Figure 2k. (b) Western blot in Figure 4a. The membrane was cut as indicated by the continuous line and probed with the indicated antibodies. Asterisks indicate unspecific bands. (c) Western blot in Figure 3e. (d) Western blot in Figure 3f. [file elife-29241-supp5.pdf]

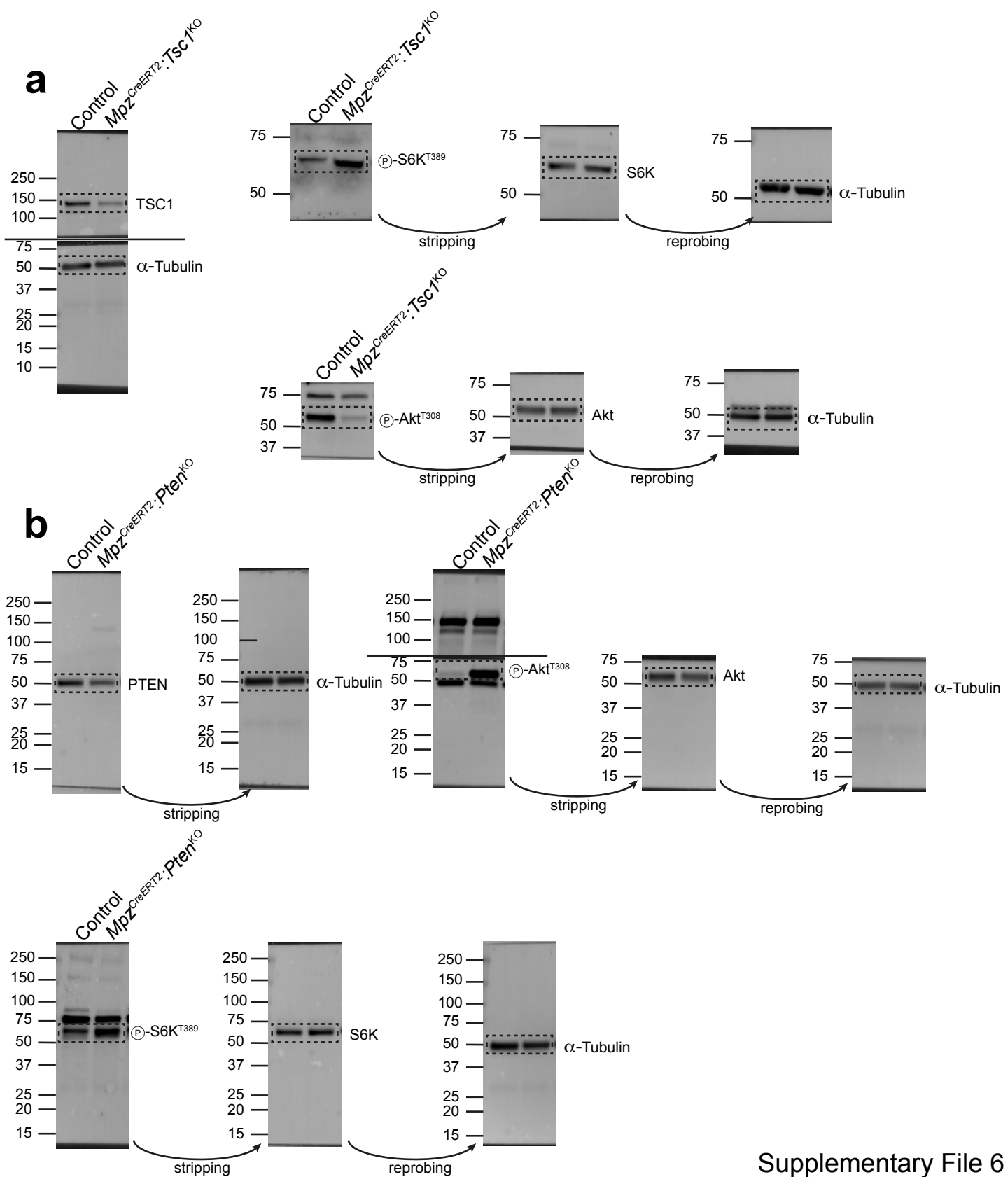

Supplement: Supplementary file 6. — (a) Western blot in Figure 5a. The membrane was cut as indicated by the continuous line and probed with the indicated antibodies. (b) Western blot in Figure 5b. The membrane was cut as indicated by the continuous line and probed with the indicated antibodies. [file elife-29241-supp6.pdf]

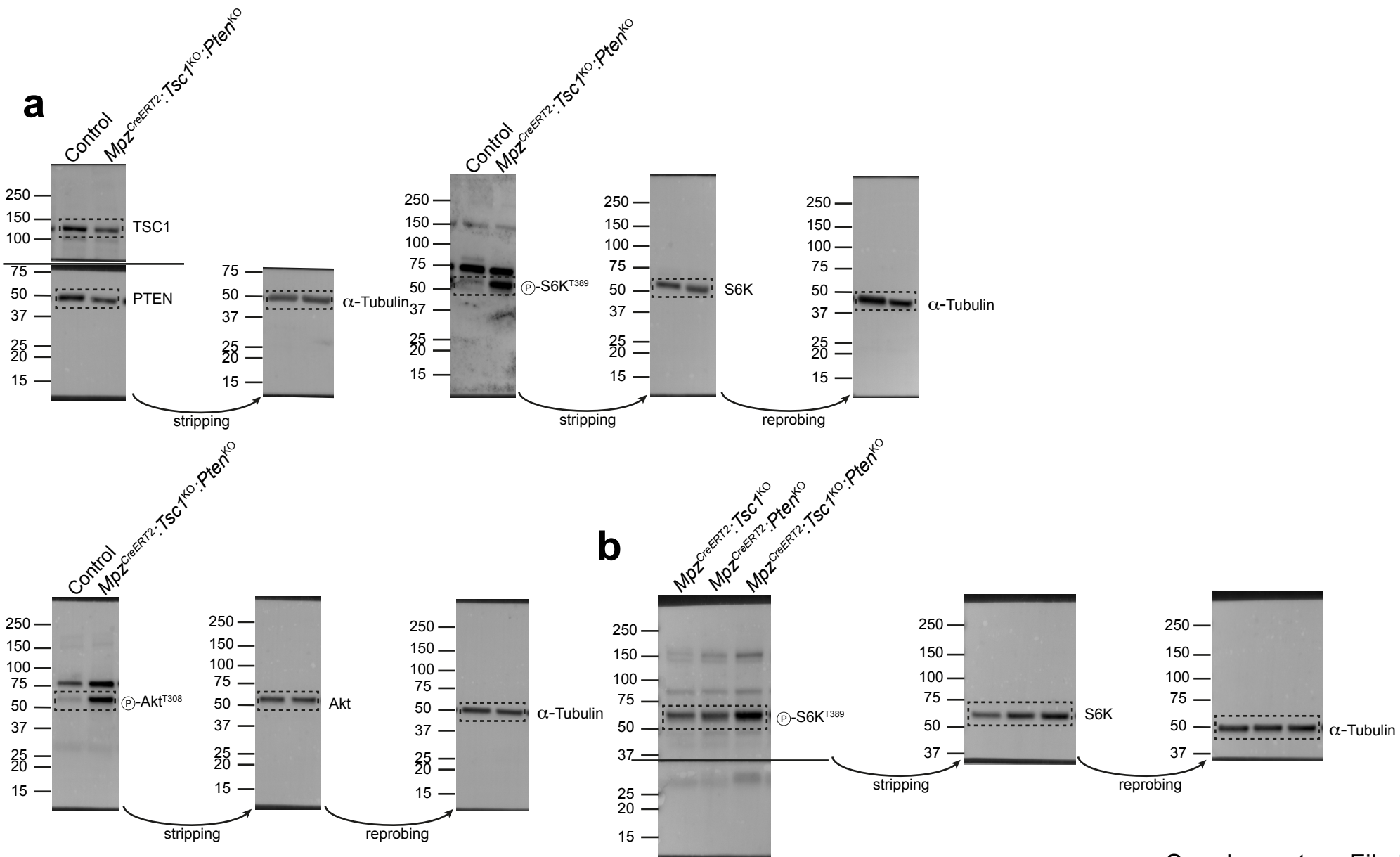

Supplement: Supplementary file 7. — (a) Western blot in Figure 5c. The membrane was cut as indicated by the continuous line and probed with the indicated antibodies. (b) Western blot in Figure 5d. The membrane was cut as indicated by the continuous line and probed with the indicated antibodies. [file elife-29241-supp7.pdf]

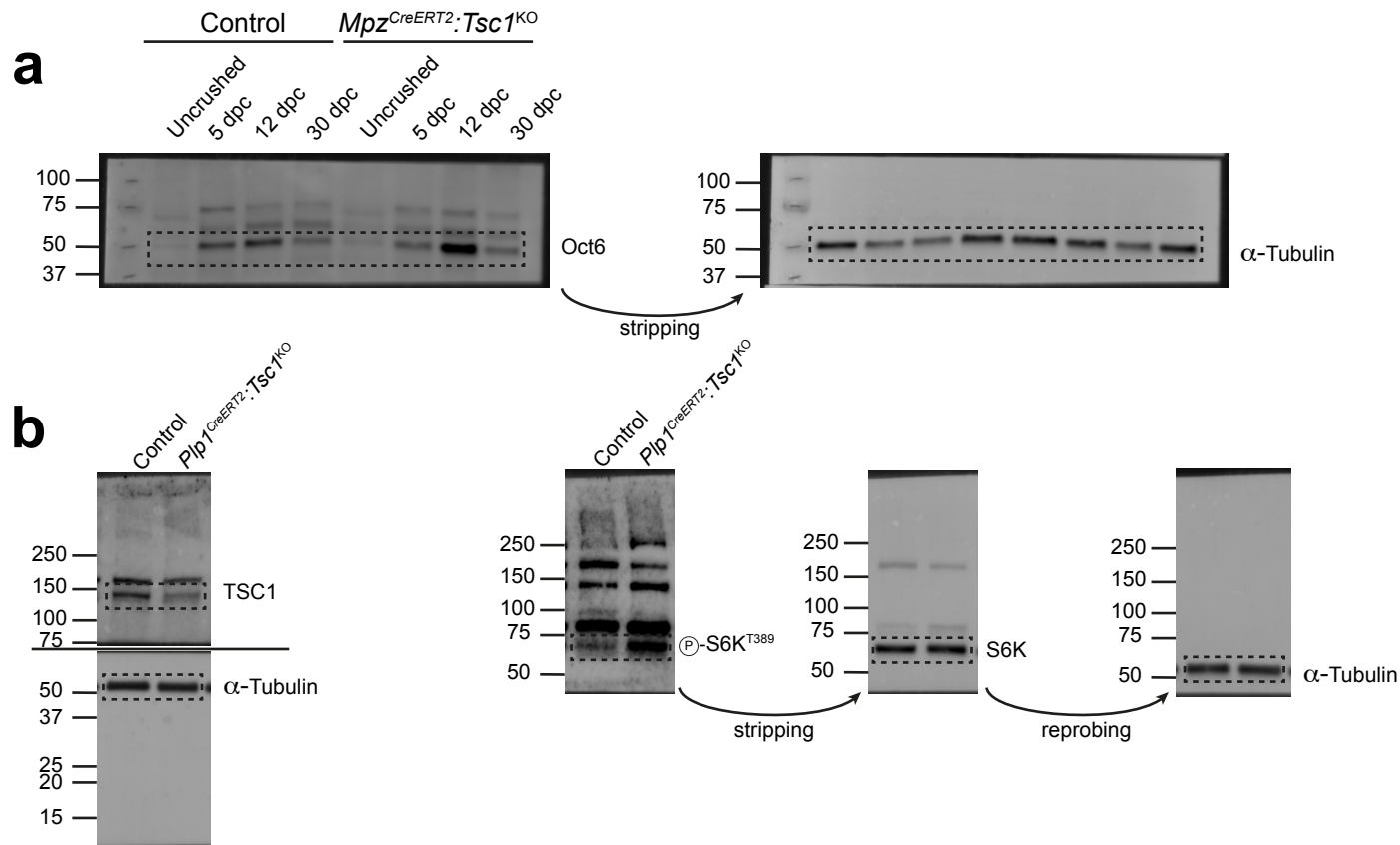

Supplement: Supplementary file 8. — (a) Western blot in Figure 6h. (b) Western blot in Figure 6j. The membrane was cut as indicated by the continuous line and probed with the indicated antibodies. [file elife-29241-supp8.pdf]
